# Sutureless repair of inferior vena cava injury using an elastomeric sealant in a mouse model with histopathological analysis of vascular healing

**DOI:** 10.1016/j.jvssci.2026.100438

**Published:** 2026-08-10

**Authors:** Yoshinori Nakahara, Shin-ichiro Ohno, Tomohiro Iwakura, Koji Fujita, Shouichirou Mineo, Masahiko Kuroda

**Affiliations:** a Department of Molecular Pathology, Tokyo Medical University, Tokyo, Japan; b Department of Cardiovascular Surgery, Sakakibara Heart Institute, Tokyo, Japan

**Keywords:** Inferior vena cava, Surgical hemostasis, Tissue adhesives, Wound healing, Mice

## Abstract

**Objective:**

To evaluate the feasibility of sutureless repair for inferior vena cava (IVC) injury using an elastomeric sealant (Hydrofit; Sanyo Chemical Industries) and to characterize the histopathological pathway of vascular healing in a mouse model.

**Methods:**

Nineteen male C57BL/6 mice underwent laparotomy under general anesthesia. A longitudinal incision exceeding 1 mm was made in the infrarenal IVC, and hemostasis was achieved using Hydrofit-applied sheets with 2 minutes of compression. The primary end point was immediate hemostatic success rate with exact 95% confidence intervals (CIs). Surviving mice were distributed across the three time points (n = 6, 6, and 6 at days 5, 10, and 30, respectively) by random allocation; one mouse at day 30 was excluded from histopathological scoring owing to inability to reliably identify the incision site. CD31 immunostaining was performed to assess endothelial coverage at the repair site. Findings were scored using a semiquantitative system modified from the Histopathological Evaluation of Acute and Long-Term Healing Scores for Advancing Wound Healing Science-Acute scale, with comparisons among time points using the Kruskal-Wallis test.

**Results:**

Immediate hemostasis was achieved in all 19 mice (100%; 95% CI, 82.4%-100%). One mouse died on postoperative day 2; anesthesia-related complications were the most likely cause, but because necropsy was not performed, procedure-related hemorrhage could not be definitively excluded, an unavoidable limitation of the primary and survival outcome estimates. The overall survival rate was 94.7% (18/19; 95% CIs, 74.0%-99.9%). No intra-abdominal adhesions were observed. Histopathological scoring demonstrated significant progression through wound healing phases (*P* = .001), with total scores increasing from 4.7 ± 1.2 at day 5 (inflammatory phase) to 7.7 ± 0.8 at day 10 (proliferative phase) and 11.4 ± 1.8 at day 30 (early maturation phase). CD31 (platelet endothelial cell adhesion molecule-1) immunostaining demonstrated CD31-positive cells at the luminal surface at all three time points; a morphologically confirmed endothelial monolayer on hematoxylin and eosin and Elastica van Gieson staining was identified at days 10 and 30, whereas elastic lamina regeneration was not observed.

**Conclusions:**

Sutureless repair using an elastomeric sealant is feasible for IVC injury in this mouse model, achieving reliable hemostasis. Although the initial wound healing response follows a physiological inflammatory-proliferative-maturation sequence culminating in endothelial coverage established by day 10, the late process is accompanied by material-driven fibrous encapsulation and intimal hyperplasia at the repair site.

**Clinical relevance:**

Venous injuries are challenging to repair due to the fragile vessel wall. This study provides histopathological evidence that sutureless repair using an elastomeric sealant facilitates initial vascular healing with endothelial coverage by day 10, although long-term material-driven responses such as fibrous encapsulation and intimal hyperplasia occur. These preclinical findings provide a mechanistic rationale for the further development of sutureless techniques for managing venous hemorrhage, particularly in situations where conventional suturing is technically difficult or time-consuming, such as during minimally invasive surgery or damage control procedures, but require confirmation in larger animal and clinical studies before clinical application.

**Article Highlights**

• **Type of Research:** Basic Science
• **Key Findings:** Sutureless repair using an elastomeric sealant achieved immediate hemostasis in all 19 mice with inferior vena cava injury (100%; 95% confidence interval, 82.4%-100%), with vessel patency maintained in all evaluated mice without intraluminal thrombus. Histopathological analysis demonstrated that although initial vascular wound healing following sutureless repair progresses through a physiological inflammatory-proliferative-maturation sequence with endothelial coverage established by day 10, the late process was accompanied by material-driven fibrous encapsulation and intimal hyperplasia.
• **Take Home Message:** Sutureless repair with an elastomeric sealant achieves reliable venous hemostasis and endothelial coverage by day 10, supporting its potential as an alternative hemostatic strategy for venous injuries. However, it induces material-driven fibrous encapsulation and intimal hyperplasia in the late process.

Venous injuries, particularly those involving major vessels such as the inferior vena cava (IVC), pose significant clinical challenges. Reported mortality rates for retrohepatic and suprarenal IVC injuries ranging from 65% to 90%, whereas infrarenal IVC injuries carry comparatively lower but still substantial mortality rates ranging from 15% to 35%,^1^ reflecting the extreme technical difficulty of hemorrhage control in a low-pressure, thin-walled system.^1^, ^2^, ^3^ In both trauma and elective surgical settings, including gynecological and oncological procedures, major venous hemorrhage remains a leading cause of intraoperative death.^3^^,^^4^ The thin and fragile nature of venous walls makes suture repair technically demanding, often requiring advanced surgical skills and prolonged operative time, during which ongoing blood loss further compromises the patient's hemodynamic status.^1^

Currently available topical hemostatic agents—fibrin sealant (Beriplast, CSL Behring GmbH; Bolheal, KM Biologics Co, Ltd), oxidized regenerated cellulose (Surgicel, Ethicon, Inc), and fibrinogen- and thrombin-coated collagen patches (TachoSil, Baxter International Inc)—have demonstrated efficacy for diffuse oozing and minor bleeding but remain inadequate for controlling active hemorrhage from larger venous defects.^5^^,^^6^ This inadequacy stems primarily from their reliance on an intact coagulation cascade and their limited physical sealing capacity against moderate-to-high flow venous bleeding. Consequently, an unmet clinical need exists for hemostatic strategies that can achieve rapid and reliable sealing of venous defects without suturing and independent of coagulation status.

Hydrofit (Sanyo Chemical Industries) is an elastomeric sealant composed of a reactive fluorine-containing polyether polyurethane prepolymer with isocyanate groups. Unlike conventional fibrin-based or cellulose-based agents, Hydrofit reacts rapidly with blood and tissue moisture to form a strong, flexible adhesive membrane independent of the coagulation cascade.^7^ This mechanism is particularly advantageous in patients with coagulopathy or those receiving anticoagulation therapy. Previous animal studies have demonstrated both the hemostatic efficacy and long-term vascular biocompatibility of this sealant: complete hemostasis was achieved in canine carotid anastomoses under full heparinization with vessel patency confirmed at 16 months,^8^ and no aortic wall thinning or tissue necrosis was observed in a rabbit model at 3-month follow-up compared with rigid adhesives.^9^ Hydrofit is clinically used in Japan for supplementary hemostasis at vascular anastomotic sites during cardiovascular surgery.^10^

Building on this preclinical and clinical foundation, previous studies have demonstrated the feasibility of sutureless repair using Hydrofit for venous bleeding in various settings, including IVC injury in a porcine model,^11^ coronary sinus rupture during cardiac surgery,^12^ and iliac vein injury.^13^ However, none of these studies characterized the biological mechanisms underlying vascular tissue repair following sutureless hemostasis. Understanding the histopathological healing process is essential for establishing the long-term safety and efficacy of this technique, and for identifying potential risks such as intimal stenosis, pseudoaneurysm formation, or abnormal inflammatory responses.

The purpose of this study was to evaluate the feasibility of sutureless repair for IVC injury using Hydrofit and to characterize the histopathological pathway of vascular healing in a mouse model. The mouse model was specifically selected for this mechanistic study because of its genetic homogeneity, the availability of well-established immunohistochemical and histopathological scoring tools, and the existence of comprehensive baseline vascular data for the C57BL/6 strain.^14^ This model complements the larger animal and clinical feasibility data and provides a foundation for future molecular and immunological investigation of vascular wound repair after sutureless hemostasis.

## Methods

### Ethical approval and study design

This study was approved by the Institutional Animal Care and Use Committee of the Tokyo Medical University (Protocol No. R7-137, approved May 14, 2025). All procedures were performed in accordance with the *Guide for the Care and Use of Laboratory Animals* (National Research Council, 2011). This study followed the Animal Research: Reporting of In Vivo Experiments guidelines for reporting animal research. Only male mice were used in this study, as described further; the decision to exclude females was made for scientific reasons and is acknowledged as a limitation with respect to generalizability.

### Sample size determination

This was an exploratory feasibility study designed to estimate the hemostatic success rate with sufficient precision. Sample size was determined on the basis of lower bound of the expected 95% confidence interval (CI) rather than statistical power for hypothesis testing. Using the Clopper-Pearson exact method, a sample size of 19 animals was selected to provide a 95% CI lower bound of at least 82% if all procedures achieved hemostasis (expected 95% CI, 82.4%-100%), ensuring an adequate margin above 80%, the prespecified clinically meaningful threshold for this initial feasibility assessment. This precision was considered sufficient for initial feasibility assessment.

### Animals

Male C57BL/6J mice (10 weeks old, body weight 24-26 g) were obtained from a commercial breeder (Japan SLC Inc) and housed at the Disease Model Research Center of the Tokyo Medical University under standard conditions (12-hour light/dark cycle, 22 ± 2 °C, 50% ± 10% humidity) with ad libitum access to food and water. Male mice were selected to reduce sex-related anatomical and body-weight variability in this initial feasibility study. Studies that involve only one sex require acknowledgment that findings may not extrapolate to the other sex; female mice were not included in this initial feasibility study.

### Anesthesia protocol

General anesthesia was induced using a combination of three agents administered intraperitoneally: medetomidine (0.75 mg/kg), midazolam (4 mg/kg), and butorphanol (5 mg/kg). At the completion of surgery, atipamezole (0.75 mg/kg) was administered intraperitoneally to reverse medetomidine.

### IVC injury model

The mean cross-sectional area of the infrarenal IVC in male C57BL/6 mice has been reported as 0.4 ± 0.1 mm^2^, corresponding to an estimated diameter of approximately 0.71 mm.^14^ To simulate major venous hemorrhage that would be challenging to control by suture repair, the incision length was standardized to exceed 1 mm, which is larger than the vessel diameter. The Hydrofit carrier sheet (approximately 5 × 5 mm) was precoated with sealant in a sufficient amount to uniformly cover the sheet surface using a syringe.

### Surgical procedure

A midline laparotomy was performed through a right paramedian skin incision. The peritoneum was carefully opened, abdominal organs were gently eviscerated and protected with saline-moistened gauze, and the infrarenal IVC was exposed between the renal veins and the iliac bifurcation. A longitudinal incision exceeding 1 mm was made on the anterior IVC wall using a microsurgical blade, creating active venous hemorrhage. The precoated carrier sheet was immediately placed over the bleeding site with the sealant surface facing the vessel wall, and gentle digital compression was maintained for 2 minutes. The compression force was manually applied to maintain vessel patency while ensuring adequate tissue contact for sealant adhesion. Hemostatic success was defined as complete cessation of bleeding at the end of the 2-minute compression period. After confirming hemostasis, abdominal organs were returned to their anatomical positions and the abdominal wall was closed in two layers using 5-0 polypropylene sutures or wound clips. All surgical procedures were performed by a single experienced cardiovascular surgeon (Y.N.) to minimize interoperator variability. All mice were monitored until recovery from anesthesia.

### End points and postoperative evaluation

The primary end point was immediate hemostatic success, defined as complete cessation of bleeding after 2 minutes of compression. Secondary end points included postoperative survival and histopathological findings. Mice were monitored daily for signs of distress, weight loss (>10% from baseline), or decreased activity. Humane end points included uncontrollable hemorrhage, signs of severe infection, or significant deterioration in general condition. Euthanasia was performed by cervical dislocation.

Surviving mice were randomly allocated to euthanasia at postoperative days 5, 10, or 30. Observation time points were selected based on the classical phases of wound healing^15^^,^^16^: day 5 was chosen to capture the inflammatory phase with early proliferative overlap, day 10 to evaluate progression through the proliferative phase, and day 30 to assess the early maturation/remodeling phase. At necropsy, the abdominal cavity was examined for evidence of rebleeding, hematoma formation, or adhesions. The IVC segment from the renal veins to the iliac bifurcation was harvested en bloc with surrounding tissue for histological examination.

### Histopathological analysis

Harvested specimens were fixed in 10% neutral buffered formalin for 24 hours, embedded in paraffin, and sectioned at 4 μm. Serial sections were stained with hematoxylin and eosin (H&E) for general morphology and Elastica van Gieson (EVG) for elastic fibers (*black*) and collagen (*red*). Immunohistochemical staining for CD31 was performed using a rabbit polyclonal antibody (Abcam; Cat. No. ab28364; 1:50) with heat-induced antigen retrieval in citrate buffer (pH 6.0). The native IVC endothelium served as an internal positive control. The anatomical location of CD31-positive cells within the repair site was determined by direct correlation with the corresponding H&E and EVG-stained serial sections; the incision site was identified by disruption of the elastic lamina on EVG staining, which allowed localization of the luminal surface. Endothelial coverage was identified when a continuous monolayer of flattened cells with elongated nuclei lined the luminal surface on both H&E and EVG staining—contiguous with the adjacent native endothelium and distinct from rounded inflammatory cells and anucleate platelet aggregates—and the corresponding site was CD31-positive. Mice in which the incision site could not be reliably identified were excluded from histopathological scoring.

Histopathological findings were evaluated using a semiquantitative scoring system modified from the Histopathological Evaluation of Acute and Long-Term Healing Scores for Advancing Wound Healing Science–Acute (HEALS-A) scale, which was originally developed for murine cutaneous wound healing assessment.^17^ The system was adapted for vascular wound healing and included four scored parameters: neutrophil infiltration, fibroblast accumulation, small vessel proliferation, and collagen deposition. Each parameter was scored from 1 to 4 (Table I). The scale was intentionally oriented so that a higher score denotes a more advanced stage of healing for every parameter: higher scores indicate greater fibroblast accumulation, small vessel proliferation, and collagen deposition, whereas for neutrophil infiltration a higher score indicates less infiltration, reflecting resolution of acute inflammation. This orientation allows the four parameters to be summed into a composite total score that increases with healing. As the HEALS-A scale was originally validated for cutaneous wounds, this adapted vascular version has not been formally validated. Intimal hyperplasia was assessed separately as a binary outcome (present or absent) owing to its dichotomous nature and complete separation across time points (absent at days 5 and 10, present at day 30) and analyzed independently using Fisher-Freeman-Halton exact test. Total score represents the sum of the four scored parameters (maximum 16 points). All histopathological evaluations were performed by a single investigator blinded to the time point.

**Table I:** Histopathological scoring system for vascular wound healing [modified Histopathological Evaluation of Acute and Long-Term Healing Scores for Advancing Wound Healing Science–Acute (HEALS-A) scale]

Parameters	Score 1	Score 2	Score 3	Score 4
Neutrophil infiltration	Abundant (+++)	Moderate (++)	Slight (+)	Absent
Fibroblast accumulation	Absent	Slight (+)	Moderate (++)	Abundant (+++)
Small vessel proliferation	Absent	Slight (+)	Moderate (++)	Abundant (+++)
Collagen deposition	<25%	25%-50%	50%-75%	75%-100%
Intimal hyperplasia	Absent	Mild (+)	Moderate (++)	Prominent (+++)

For neutrophil infiltration, higher scores indicate less cellular density (resolution of inflammation). In contrast, for fibroblast accumulation and small vessel proliferation, higher scores indicate greater cellular density (increase in proliferative cells), consistent with more advanced healing. For collagen deposition, higher scores indicate greater percentage area coverage. Intimal hyperplasia was assessed separately as a binary outcome (present/absent) owing to its bimodal temporal distribution and is not included in the total score. The scale is intentionally oriented so that a higher score reflects a more advanced stage of healing for every parameter (for neutrophil infiltration, this corresponds to less cellular density). This adapted vascular version of the HEALS-A scale has not been formally validated for vascular wound healing and should be regarded as an internally consistent, semiquantitative index.

### Statistical analysis

The hemostatic success rate and survival rate were each calculated as proportions with exact 95% CIs computed using the Clopper-Pearson method.

Histopathological scores for each parameter were compared among the three time points (days 5, 10, and 30) using the Kruskal-Wallis test. When significant differences were detected, post hoc pairwise comparisons were performed using the Steel-Dwass test. Data are presented as means ± standard deviation. Intimal hyperplasia presence was compared across time points using Fisher-Freeman-Halton exact test. A *P* value of <.05 was considered statistically significant. Given the exploratory nature of this study and the small sample size at each time point (n = 5-6), results should be interpreted as hypothesis-generating; effect sizes and CIs are not reported for histological parameters, which is acknowledged as a limitation. All analyses were performed using R version 4.3.0 (R Foundation for Statistical Computing).

## Results

### Hemostatic efficacy and survival

A total of 19 male C57BL/6 mice underwent the sutureless repair procedure. Immediate hemostasis, defined as complete cessation of bleeding after 2 minutes of compression, was achieved in all 19 mice (100%; 95% CI, 82.4%-100%). No intraoperative deaths or episodes of uncontrollable bleeding occurred.

One mouse was found dead on postoperative day 2. This mouse exhibited markedly reduced spontaneous activity and prolonged sedation during the recovery period; since complete hemostasis had been confirmed, and no wound bleeding, abdominal distension, or other signs suggestive of intra-abdominal hemorrhage were identified, conservative monitoring was continued. Necropsy was not performed and the cause of death was most likely attributable to anesthesia-related complications based on clinical observations, although procedure-related hemorrhage cannot be definitively excluded in the absence of necropsy. The absence of a confirmatory necropsy is an unavoidable limitation that precludes definitive attribution of this single death and introduces uncertainty into both the primary hemostatic and the survival outcome estimates. The remaining 18 mice survived to their scheduled euthanasia time points, yielding an overall survival rate of 94.7% (18/19; 95% CI, 74.0%-99.9%). Surviving mice were distributed across the three time points (n = 6, 6, and 6 at days 5, 10, and 30, respectively) by random allocation.

### Macroscopic findings at necropsy

Macroscopic findings at each time point are summarized in Table II. At day 5, Hydrofit remained loosely adherent to the IVC in three mice and firmly adherent in the remaining three; a small hematoma was present in one mouse but did not compromise the repair site. By day 10, Hydrofit remained adherent to the IVC repair site; in four of six mice, the sealant was surrounded by newly formed tissue and remained isolated from adjacent abdominal organs without adhesion formation. No hematoma was identified in any mouse at this time point. At day 30, a pseudotumor-like fibrous mass—representing organized fibrous encapsulation of the residual Hydrofit—was observed; the Hydrofit had become completely detached from the IVC and was displaced from the original repair site. Notably, no intra-abdominal adhesions were identified at days 5 or 10, and only one mouse at day 30 exhibited a localized adhesion that did not involve adjacent bowel.

**Table II:** Macroscopic findings at necropsy

Time points	n	Complete hemostasis	Sealant adherence	Hematoma/adhesions
Day 5	6	6/6 (100)	Loose: 3, adherent: 3	Hematoma: 1 present, 5 absent; adhesions: none
Day 10	6	6/6 (100)	Loose: 2, mass: 4	Hematoma: none; adhesions: none
Day 30	6	6/6 (100)	Mass: 5, flat: 1	Hematoma: none; adhesions: localized (1 mouse)

Values expressed as n (%) or descriptive categories. Sealant adherence categorized as: Loose = sheet mobile with no fixation; Adherent = sheet fixed to vessel wall; Mass = organized fibrous nodular mass formation; Flat = incorporated into vessel wall surface.

### IVC patency

Histopathological cross-sections of the IVC demonstrated a patent lumen without intraluminal thrombus in all 18 evaluated mice across the three time points. There was no evidence of luminal stenosis attributable to the repair. The IVC wall adjacent to the repair site maintained its structural integrity throughout the observation period.

### Histopathological scoring

Histopathological scores at each time point are summarized in Table III. At day 30, one mouse was excluded from histopathological scoring because the IVC incision site could not be reliably identified on serial sections, resulting in n = 5 for this time point. Total scores demonstrated significant progressive increase across the three time points (*P* = .001, Kruskal-Wallis test), indicating orderly advancement through wound healing phases.

**Table III:** Histopathological scoring of vascular wound healing at each time point

Parameters	Day 5 (N = 6)	Day 10 (N = 6)	Day 30 (N = 5)	*P* value
Neutrophil infiltration	1.0 ± 0.0	1.3 ± 0.5	2.0 ± 1.0	.081
Fibroblast accumulation	1.0 ± 0.0	1.3 ± 0.5	2.6 ± 0.5	.003
Small vessel proliferation	1.2 ± 0.4	2.3 ± 0.5	2.8 ± 0.4	.003
Collagen deposition	1.5 ± 0.8	2.7 ± 0.5	4.0 ± 0.0	.001
Total score	4.7 ± 1.2	7.7 ± 0.8	11.4 ± 1.8	.001
Intimal hyperplasia^a^	0/6 (0%) [0%-39.3%]	0/6 (0%) [0%-39.3%]	4/5 (80%) [28.4%-99.5%]	.002^a^

Values are means ± SD for scored parameters, or n/N (%) [95% CI] for intimal hyperplasia. Total score is the sum of four parameters (such as neutrophil infiltration, fibroblast accumulation, small vessel proliferation, and collagen deposition; maximum 16 points). *P* values for scored parameters calculated using the Kruskal-Wallis test.

a Intimal hyperplasia assessed as a binary outcome; *P* value by Fisher-Freeman-Halton exact test; 95% CIs calculated using the Clopper-Pearson exact method. Higher total score indicates more advanced wound healing.

**Fig 1:**
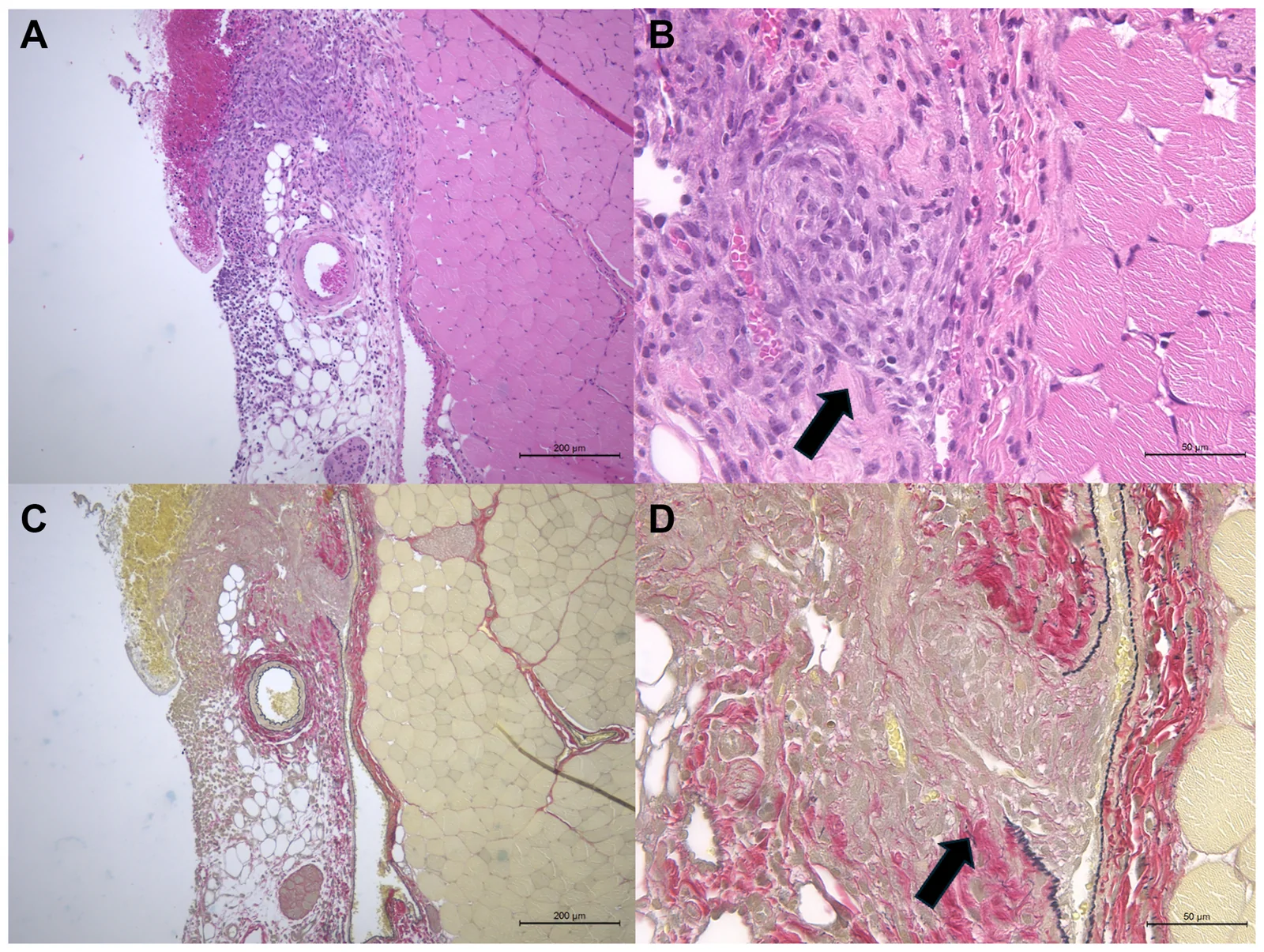
Histological evaluation of the inferior vena cava (IVC) 5 days after sutureless repair. **A,** Low-magnification hematoxylin and eosin (H&E) staining showing a patent IVC lumen without intraluminal thrombus. **B,** High-magnification H&E. The repair site (*arrow*) is bridged by a cellular barrier predominantly composed of neutrophils, forming a hemostatic seal in the absence of the native vessel wall structure. **C,** Low-magnification Elastica van Gieson (EVG) staining. **D,** High-magnification EVG. The incision site (*arrow*) lacks elastic fibers; the black-staining elastic lamina is intact in the adjacent native wall but absent at the repair site. Original magnification: × 10 **(A** and **C)**; × 40 **(B** and **D)**.

**Fig 2:**
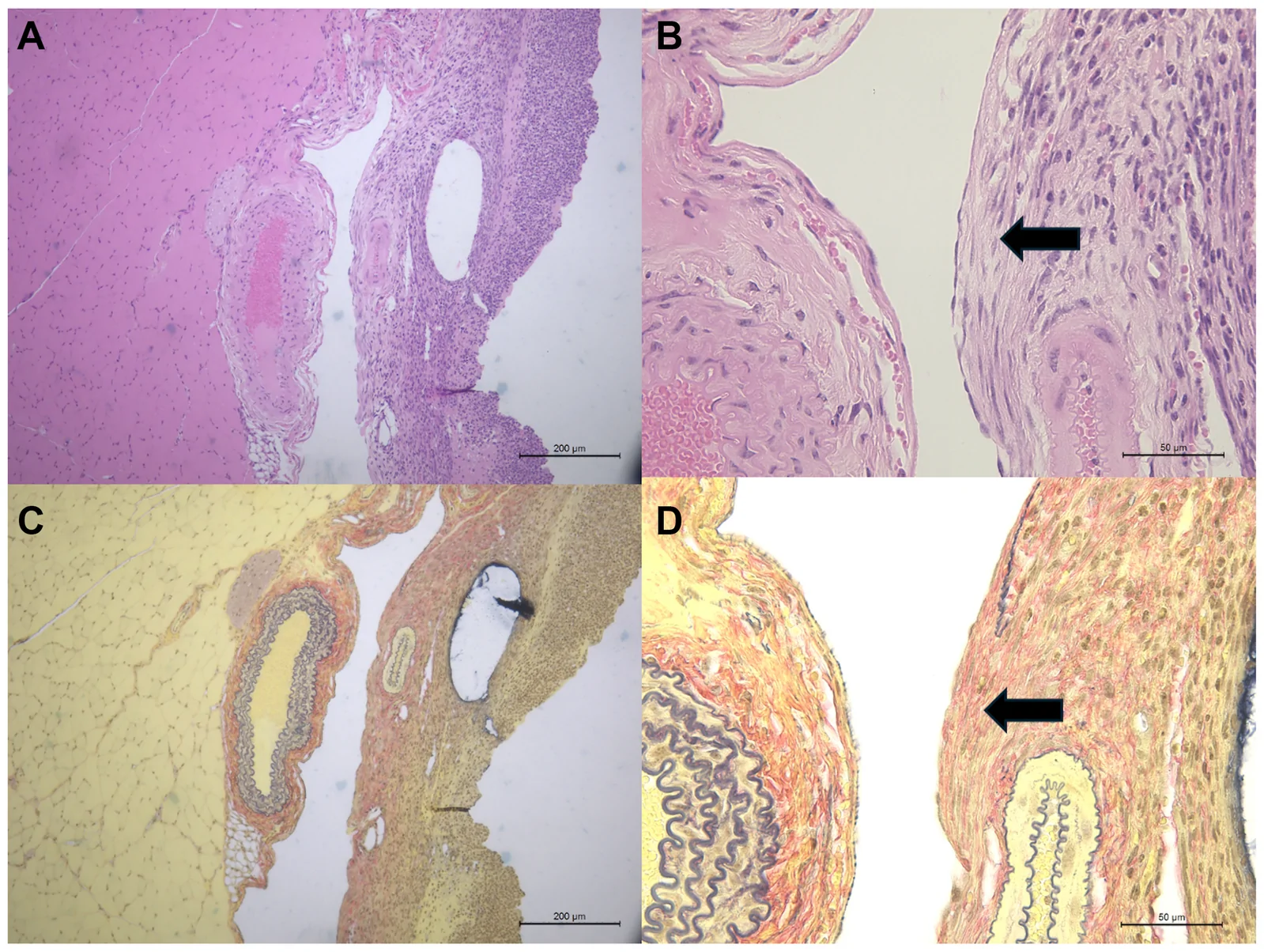
Histological evaluation of the inferior vena cava (IVC) 10 days after sutureless repair. **A,** Low-magnification hematoxylin and eosin (H&E) staining showing a patent lumen without intraluminal thrombus. **B,** Intermediate-magnification H&E. The repair site (*arrow*) contains a mixture of neutrophils, and spindle-shaped fibroblasts, indicating the transition from acute inflammation to subacute granulation tissue formation. **C,** Low-magnification Elastica van Gieson (EVG) staining. Elastic fibers (*dark purple*) delineate the intact wall from the repair site. **D,** Intermediate-magnification EVG. The incision site (*arrow*) lacks elastic lamina regeneration but shows early fibrous matrix deposition replacing granulation tissue. The luminal surface shows a flattened endothelial monolayer **(B** and **D)**. Original magnification: × 10 **(A** and **C)**; × 40 **(B** and **D)**.

**Fig 3:**
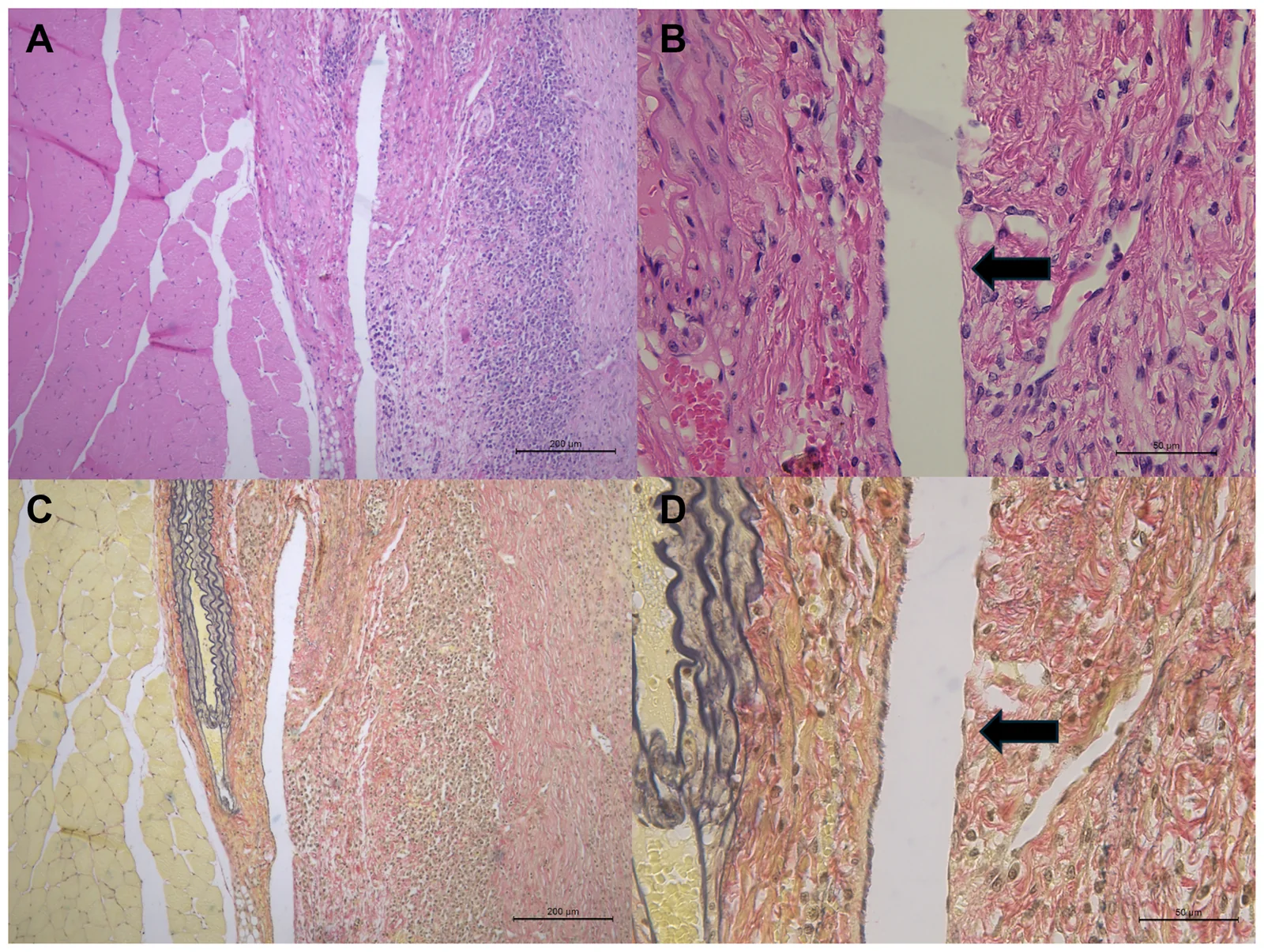
Histological evaluation of the inferior vena cava (IVC) 30 days after sutureless repair. **A,** Low-magnification hematoxylin and eosin (H&E) staining showing a patent lumen without intraluminal thrombus. **B,** Intermediate-magnification H&E. The repair site contains mature fibrous scar tissue with spindle-shaped fibroblasts and dense collagen fibers. Prominent intimal hyperplasia is observed on the luminal surface. The *arrow* indicates the incision site. **C,** Low-magnification Elastica van Gieson (EVG) staining showing abundant collagen (*red*) at the repair site and well-preserved elastic lamina (*dark purple/black*) in the adjacent native wall. **D,** High-magnification EVG. Dense mature collagen fibers are present at the repair site without elastic lamina regeneration. A flattened endothelial monolayer is apparent on the luminal surface. The *arrow* indicates the incision site. Original magnification: × 10 **(A** and **C)**; × 40 **(B** and **D)**.

At day 5, low-magnification view demonstrated a patent IVC lumen without intraluminal thrombus (Fig 1, *A*), and higher magnification revealed abundant neutrophil infiltration (score 1.0 ± 0.0) forming a cellular barrier at the repair site (Fig 1, *B*). EVG staining showed disrupted elastic lamina at the incision site against the intact adjacent native wall (Fig 1, *C* and *D*). Minimal fibroblast accumulation (1.0 ± 0.0) and early small vessel proliferation (1.2 ± 0.4) were observed, consistent with the inflammatory phase. By day 10, the lumen remained patent without intraluminal thrombus (Fig 2, *A*), and intermediate-magnification view showed a mixture of neutrophils and spindle-shaped fibroblasts at the repair site, indicating transition from acute inflammation to subacute granulation tissue formation (Fig 2, *B*). EVG staining demonstrated absent elastic lamina regeneration at the incision site with early fibrous matrix deposition replacing the granulation tissue (Fig 2, *C* and *D*). Neutrophil infiltration remained elevated (1.3 ± 0.5), while small vessel proliferation and collagen deposition increased progressively (2.3 ± 0.5 and 2.7 ± 0.5, respectively), representing the transition into the proliferative phase. At day 30, the lumen remained patent without intraluminal thrombus (Fig 3, *A*), and intermediate-magnification view revealed mature fibrous scar tissue containing spindle-shaped fibroblasts and dense collagen fibers, with prominent intimal hyperplasia on the luminal surface (Fig 3, *B*). EVG staining showed abundant collagen at the repair site with well-preserved elastic lamina in the adjacent native wall (Fig 3, *C*), and high-magnification view confirmed dense mature collagen fibers without elastic lamina regeneration, whereas a flattened endothelial monolayer was apparent on the luminal surface (Fig 3, *D*). Collagen deposition reached maximal scores (4.0 ± 0.0), and fibroblast accumulation increased further (2.6 ± 0.5), indicating entry into the early maturation phase. Statistical comparisons of individual parameters among the three time points are summarized in Table III. Notably, intimal hyperplasia was observed in four of five mice at day 30 (80%; 95% CI, 28.4%-99.5%; *P* = .002, Fisher-Freeman-Halton exact test), representing a statistically significant trend compared with no cases at days 5 or 10 (0%; 95% CI, 0%-39.3% for each time point). In contrast, elastic lamina integrity remained disrupted at the repair site in all sections, with no evidence of regeneration at any time point.

**Fig 4:**
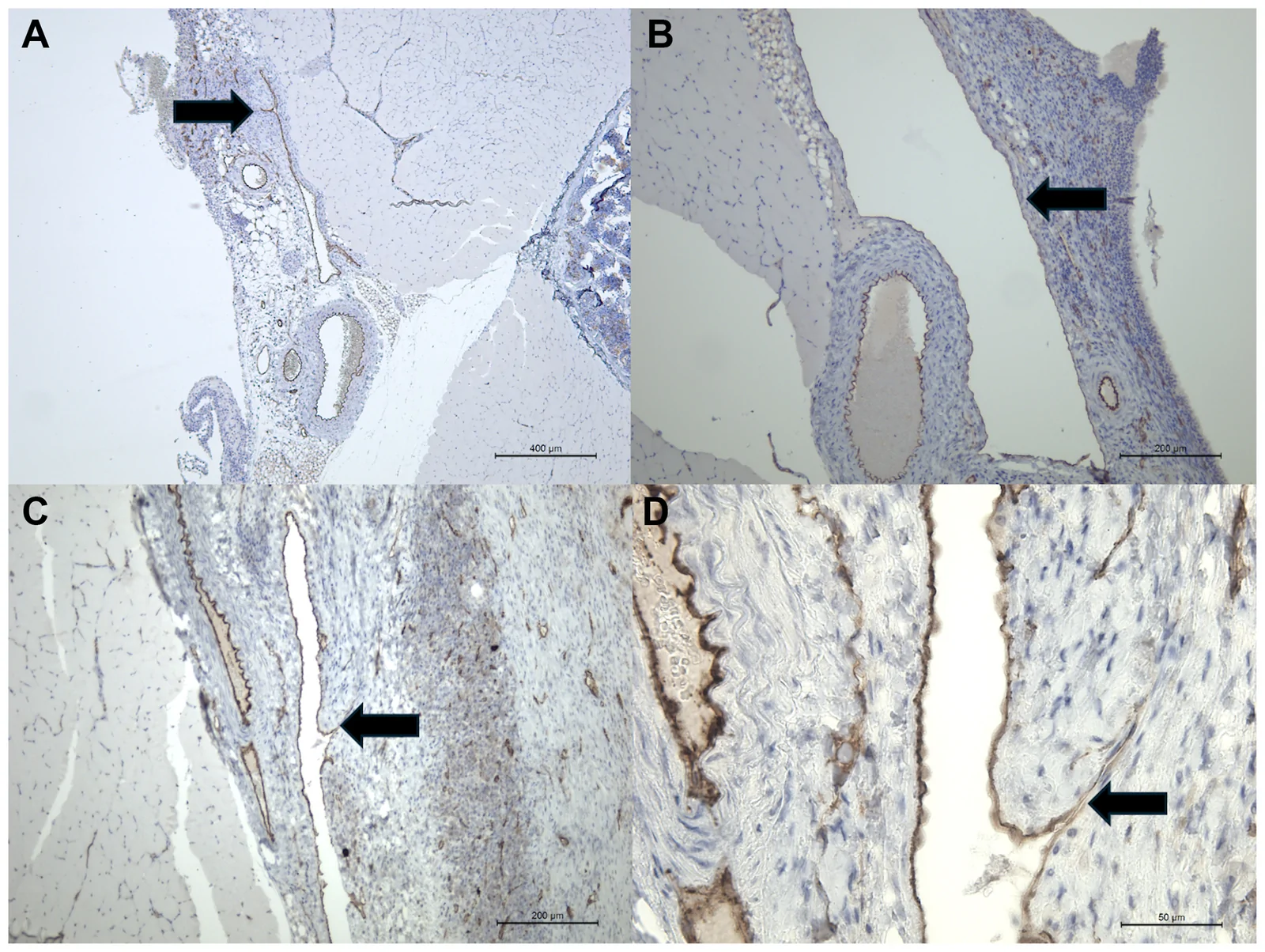
CD31 immunostaining. CD31-positive cells (*brown*) line the luminal surface of the repair site (*arrowhead*) at all three time points; at days 10 and 30 these correspond to a morphologically identified endothelial monolayer (Figs 2 and 3). Infrarenal inferior vena cava at 5 days after sutureless repair **(A)**, 10 days **(B)**, 30 days **(C** and **D)**. Original magnification: × 5 **(A)**; × 10 **(B** and **C)**; × 40 **(D)**.

CD31-positive cells were present at the luminal surface of the repair site at all three time points (days 5, 10, and 30; Fig 4, *A-D*). At days 10 and 30, a morphologically identifiable endothelial monolayer was evident on both H&E and EVG staining (Figs 2, *B* and *D* and 3, *B* and *D*) and corresponded to the CD31-positive lining. At day 5, in contrast, CD31-positive cells were present, but a definitive endothelial monolayer was not identifiable; given CD31 expression by platelets and inflammatory cells, the day-5 signal cannot be attributed to functional re-endothelialization. Endothelial coverage was thus established by day 10 and maintained throughout the observation period.

Neutrophil infiltration did not show statistically significant differences among the three time points (*P* = .081), reflecting the persistent neutrophil aggregation at the sealant-tissue interface through day 30, although a trend toward resolution was apparent (score 1.0 at day 5 vs 2.0 at day 30; higher score = less infiltration). Given the small sample size at each time point and the absence of estimated effect sizes and CIs, this nonsignificant result should be interpreted as inconclusive rather than as evidence of no change over time.

## Discussion

This study demonstrates that sutureless repair using the elastomeric sealant Hydrofit is feasible for infrarenal IVC injury in a mouse model, achieving 100% immediate hemostasis in all 19 mice. These findings extend the previous feasibility evidence from a porcine IVC model^11^ and clinical case reports^12^^,^^13^ by providing systematic histopathological characterization of the vascular healing process following sutureless repair.

IVC patency was maintained in all 18 mice evaluated histologically, with no intraluminal thrombus formation at any time point. The elastomeric properties of the sealant—particularly its capacity to deform under physiological pressure changes—may contribute to patency preservation by preventing mechanical disruption of the luminal surface during the healing period.

The histopathological analysis revealed that venous sutureless repair with Hydrofit follows the classical phases of wound healing.^15^^,^^16^ Semiquantitative scoring using the modified HEALS-A system^17^ provided objective evidence of this progression, with total scores significantly increasing from 4.7 ± 1.2 at day 5 to 7.7 ± 0.8 at day 10 and 11.4 ± 1.8 at day 30 (*P* = .001). This quantitative approach enabled standardized evaluation of vascular wound healing dynamics and demonstrated clear discrimination between healing phases. The orderly healing sequence observed—from acute neutrophilic inflammation at day 5, through fibroblastic proliferation with collagen deposition at day 10, to early scar maturation, with progressive endothelial coverage confirmed at all time points—suggests that the elastomeric sealant provides a biocompatible environment for the initial phase of physiological vascular repair without disrupting the early wound healing cascade, thereby supporting the theoretical basis for the safety of sutureless repair; as discussed below, this early physiological response is followed by a distinct, material-driven fibrous encapsulation in the late phase.

An important finding is the establishment of endothelial coverage at the repair site by day 10. Endothelial identity was established at days 10 and 30, but not at day 5, by concordant morphology and CD31 positivity (Figs 2 and 3). This observation is consistent with restoration of a functional luminal barrier by day 10 through physiological healing processes. Given that Hydrofit maintains its sealing capacity for approximately 2 to 3 months, endothelial coverage achieved within this period would be expected to ensure the long-term integrity of the repair site even after the sealant eventually detaches from the vessel wall. On the other hand, the absence of elastic lamina regeneration at day 30 also warrants attention. Unlike collagen and the endothelium, elastic fibers have limited regenerative capacity in adult mammals,^18^ a consequence of the highly specialized machinery required for tropoelastin cross-linking and elastic fiber assembly, which is predominantly active only during vascular development. Although the elastic lamina contributes to vessel wall compliance and recoil, its absence at the repair site may be functionally compensated by surrounding mature collagen and the restored endothelial barrier. In the low-pressure venous system, sutureless repair without elastic lamina regeneration may be functionally acceptable; however, long-term studies are warranted to evaluate whether this structural difference leads to clinically relevant alterations in wall compliance. Of particular note, application of this technique to the high-pressure arterial system may pose greater risks—including pseudoaneurysm formation—in the absence of elastic lamina regeneration and warrants dedicated investigation.

Persistent neutrophil aggregation at the sealant-tissue interface throughout all observation periods reflects the sustained chemotactic signal provided by the nonabsorbable sealant material, consistent with the established foreign body response in which sterile implant material triggers predictable neutrophil recruitment independent of infection.^19^ Neutrophil infiltration scores showed no statistically significant differences across time points (*P* = .081), further supporting a material-driven rather than infection-related etiology. Previous murine hemostasis experiments using a tetrapolyethylene glycol hydrogel have similarly reported inflammatory cell infiltration at the repair site,^20^ and postmarketing surveillance of Hydrofit in 542 patients across 46 Japanese institutions reported no significant increase in the incidence of infection or clinically relevant inflammation,^7^ corroborating the clinical safety of this response. Significant neutrophil accumulation has also been documented in association with high biocompatibility in other mouse implant models: VandeVord et al^21^ reported that despite large neutrophil migration into chitosan scaffold implants, there were minimal signs of material-specific inflammation. The contemporary understanding of neutrophil biology further supports a constructive interpretation: neutrophils actively orchestrate tissue repair and facilitate revascularization,^22^ whereas tissue-resident macrophages dynamically regulate neutrophil swarm formation through membrane sequestration of cellular damage signals, preventing excessive inflammatory injury.^23^ Importantly, the confirmed presence of CD31-positive endothelial cells at the luminal surface of the repair site even during this acute neutrophilic phase at day 5 suggests that the local inflammatory environment did not impair endothelial cell survival or recruitment. In the present study, neutrophil aggregation remained localized without evidence of systemic inflammation, IVC luminal compromise, or significant adhesion formation, supporting the interpretation that this response represents a species-specific chemotactic effect intrinsic to the material rather than a signal of pathological inflammation or clinical concern.

A further notable observation was the continuously increasing fibroblast accumulation across all time points (scores 1.0, 1.3, and 2.6 at days 5, 10, and 30, respectively), a trajectory that deviates from the pattern expected in physiological wound healing. In normal tissue repair, fibroblast activity peaks during the proliferative phase—approximately days 7 to 14 in murine models—and subsequently declines as extracellular matrix matures, collagen cross-links, and fibroblasts undergo apoptosis or transition to a quiescent phenotype. The progressive and unresolved fibroblast accumulation observed here is more consistent with the chronic foreign body response, in which persistent nonabsorbable implant material sustains fibroblastic recruitment as part of an adaptive encapsulation process.^24^^,^^25^ This ongoing fibroblastic activity, coupled with maximal collagen deposition at day 30 (score 4.0), most likely underlies the macroscopic formation of the pseudotumor-like fibrous mass and should be interpreted not as impaired tissue repair, but as a material-driven fibrous encapsulation distinct from physiological wound healing. Whether fibroblast accumulation reaches a plateau or gradually resolves at later time points remains unknown and warrants evaluation in long-term follow-up studies.

The mouse model offers important advantages for mechanistic studies, including genetic homogeneity, cost-effectiveness, and the availability of well-characterized immunohistochemical markers. However, several translational limitations must be acknowledged. Murine and human neutrophils differ substantially in receptor expression, granule protein content, oxidant production, and intracellular signaling pathways; studies using exclusively murine neutrophils should restrict their conclusions accordingly and should not be generalized to human neutrophil biology without validation.^26^ The small vessel dimensions and relatively lower hemodynamic demands of the mouse IVC also limit direct extrapolation of the repair mechanics to the human clinical setting. This study is intended as a complementary mechanistic investigation that builds upon and should be interpreted alongside the existing larger animal and clinical data. Third, only male mice were studied, so sex-based differences in inflammatory and healing responses mean that generalizability to females is unknown. Taken together, these interspecies and model constraints indicate that the present results establish biological feasibility and mechanism in a small-animal model but do not establish direct clinical applicability, and should be interpreted as hypothesis-generating.

This study has several limitations. First, necropsy was not performed on the mouse that died on postoperative day 2; although the clinical course was consistent with anesthetic complications, procedure-related hemorrhage cannot be definitively excluded without pathological confirmation. Second, the sample size at each time point was small (n = 5-6), limiting statistical power for detecting subtle differences in histological parameters; in particular, the nonsignificant trend in neutrophil infiltration (*P* = .081) may represent insufficient power rather than a true null effect. Accordingly, nonsignificant findings—including that for neutrophil infiltration—should not be interpreted as evidence of no difference, particularly because effect sizes and CIs were not estimated for the histological parameters. Third, the manual compression force applied during the repair procedure was not standardized or quantitatively measured, which may affect reproducibility. Fourth, the observation period was limited to 30 days; the long-term studies are required to assess the durability of the repair and the potential impact of persistent foreign-body reaction. Fifth, only male C57BL/6 mice were studied; results may not apply to female animals or other strains. Sixth, the modified HEALS-A scoring system, although adapted from a validated cutaneous scale, has not been formally validated for vascular wound healing; the composite total score should therefore be regarded as an internally consistent, semiquantitative index rather than a validated measure. Seventh, no infection-specific investigations (such as Gram staining or bacterial culture) or measurements of systemic inflammatory markers were performed; although the localized, nonprogressive inflammatory pattern without luminal compromise, adhesion, or systemic signs is most consistent with a foreign-body response, low-grade infection or chronic inflammation cannot be directly excluded.

## Conclusions

Sutureless repair using an elastomeric sealant is a feasible strategy for achieving reliable hemostasis in a mouse model of IVC injury. Although the procedure facilitates endothelial coverage by day 10 and follows an initial physiological wound healing response, it is accompanied by late material-driven fibrous encapsulation and intimal hyperplasia without elastic lamina regeneration. These findings support the further investigation of this technique for challenging venous hemorrhage. However, given the anatomical and physiological differences between the murine model and human major venous injury, they should not be extrapolated directly to clinical practice, and long-term confirmatory studies in larger animals and, ultimately, clinical settings are warranted to fully understand the clinical implications of these material-driven histological changes.

## Author Contributions

Conception and design: YN, MK

Analysis and interpretation: YN, SO, MK

Data collection: YN, SO, TI, KF, SM

Writing the article: YN

Critical revision of the article: YN, SO, TI, KF, SM, MK

Final approval of the article: YN, SO, TI, KF, SM, MK

Statistical analysis: YN

Obtained funding: YN, MK

Overall responsibility: SO

## Declaration of generative ai and ai-assisted technologies in the writing process

During the preparation of this work, the authors used DeepL for spell-checking and grammar verification. After using this service, the authors reviewed and edited the content as needed and take full responsibility for the content of the publication.

## Funding

This study was supported by the Sakakibara Internal Research Grant for Promotion of Sciences, 2025.

## Disclosures

None.
